# Enhancing community resilience in the context of trauma: The Morandi Bridge collapse in Italy

**DOI:** 10.1002/ajcp.12772

**Published:** 2024-12-22

**Authors:** Laura Migliorini, Martina Olcese, Paola Cardinali

**Affiliations:** ^1^ Department of Educational Science Genoa Italy; ^2^ Department of Human and Social Science Mercatorum University Rome Italy

**Keywords:** community resilience, community trauma, ecological maps, Morandi Bridge collapse, resilience toolkit, stakeholder analysis

## Abstract

Community resilience increases collective capacity to enact change and restore communities following trauma. Using the *Beyond the Bridge Project* as a case study following the Morandi Bridge Collapse, we conducted 10 consultations with the Project Lead Group. We employed a stakeholder analysis, and ecological maps performed using the Communities Advancing Resilience Toolkit methodology. The findings highlight the involvement and categorization of stakeholders based on their post‐trauma intervention attitudes. The ecological maps facilitated communication and community competence between different stakeholder groups, which are key aspects of community resilience. We suggest that this methodology is promising for future research in trauma‐affected communities.

## Highlights

- Stakeholder analysis and ecomaps can identify key stakeholders and understand the relationships.
- Community resilience enhances the collective capacity to influence change after trauma.
- The Communities Advancing Resilience Toolkit methodology offers a bottom‐up approach to foster community resilience

## INTRODUCTION

Communities, understood as a group of people who share the same values and interests and have similar experiences and needs, often face the challenge of coping with the consequences of a tragic and traumatic event (Mwanri et al., 2021). Community resilience theories focus on the ability of community members to cope with environmental and climate change, natural disasters and catastrophes, and negative traumatic human events such as terrorism or government mismanagement of disasters by using available resources to overcome difficulties (Kais & Islam, 2016). Members of a resilient community develop a collective capacity to influence change to sustain and renew the community and develop new pathways for the future (Baldwin et al., 2018). The adaptive capacities that lead to resilience in a community encompass many dynamic attributes, linkages, and transactional relationships, including community competence, economic development, information and communication, and social capital. In addition, political, economic, and natural forces operating at broader ecological levels influence these community‐level capacities (Norris et al., 2008).

A bottom‐up approach is one of the main drivers of community resilience, and several relevant strategies have been identified, such as empowering local people, creating place‐based solutions, improving economic growth efforts, and developing civic participation (Wu & Chen, 2023). Community resilience is therefore considered as the ability of a community to adapt to hostile situations and cope with traumatic events in innovative ways (Matthews et al., 2014). It is also understood as the ability of community members to engage in coordinated action projects within their context despite constraining events and structures (Mayer, 2019). Therefore, the focus is placed on the planning and design phase of the community that follows a critical event and prevents a possible subsequent event. Community resilience refers to the aftermath of a traumatic event and the preparation of the community for the event itself (community preparedness). Trauma refers to situations related to war, violence, disaster or sudden loss that are characterized by an extreme sense of helplessness in those involved, a change in expectations and beliefs, and with loss of control that can lead to major psychological consequences of suffering (Kleber, 2019). Community resilience can promote recovery from trauma through the enhancement of community resources and protective factors with a subsequent reduction in the negative consequences associated with the traumatic event (Kendra & Wachtendorf, 2007). In the literature, community resilience can be conceptualized as a process and as an outcome (Clark‐Ginsberg et al., 2020). However, it is difficult to provide a clear distinction between these two terms given the factors that contribute to the promotion of resilience during the process are the same as those that determine it as outcomes (Olcese et al., 2024; Rochira et al., 2023).

Community resilience is discussed in the context of change rather than static, a change that produces a state of local disorganization that leads to transformations in the functioning and identity of the community (Matarrita‐Cascante et al., 2022). In this context, resilience is understood as a continuous process of change and adaptation resulting from the need to respond to stress (Magis, 2010). Therefore, community resilience as a process refers to the dynamic and evolving ability of a community to cope, adapt and learn from challenges. It involves the active participation of community members, the development of support networks and the implementation of prevention strategies. This perspective emphasizes the fluidity of social interactions and the ability to adapt dynamically over time (Norris et al., 2008; Ross & Berkes, 2014).

On the other hand, community resilience as an outcome is the tangible expression of a community's ability to successfully cope with the negative effects of a stressful event. This can be expressed as the presence of a set of positive attributes, a wide range of skills, competencies and knowledge of a community that enable it to recover and respond to disasters (Coles & Buckle, 2004; Patel et al., 2017). When community resilience is conceptualized as a process, stakeholder involvement at all stages of the process (i.e., assessment, feedback, planning, and action) emerges as a key factor in promoting resilience (Pfefferbaum et al., 2013).

Stakeholder participation in community resilience processes offers numerous benefits, including the creation of a platform for sharing experiences and knowledge (Sharifi, 2016). Furthermore, the process of stakeholder interaction can lead to a form of collaborative social learning that enables shifts in knowledge and understanding that translate into changed policies and practices (Burnside‐Lawry & Carvalho, 2016). Indeed, it can facilitate understanding of the meaning of resilience and the community's position on resilience through stakeholder engagement characterized by trust, open dialogue, and collaborative decision‐making processes (Ashmawy, 2021; Cox & Hamlen, 2015). If the process is developed and implemented in collaboration with different stakeholders, this can strengthen their role in decision‐making processes, increase transparency in the planning phases, and promote social networking. Stakeholder involvement is therefore crucial in finding solutions to major community challenges, including problems related to disaster risk reduction (Burnside‐Lawry & Carvalho, 2016).

Community members must therefore be involved in all phases of resilience projects. Various actors such as schools, government agencies and private organizations can be considered stakeholders in these projects. To this end, it is important to understand the axes of power relations within the community among different stakeholders between the various interest groups. Power mechanisms can take many forms, for example, boundaries that limit stakeholders' options, stakeholders' ability to participate in decision‐making processes. Thus, social power relations can involve various interactions (e.g., political, social, psychological) and these influence the ability of individuals in the community to trust, communicate openly and have decision‐making power; this aspect could also influence attitudes towards engagement with others. In addition, stakeholders may have varying levels of awareness of power relations in the target community, which may influence their ability to critically evaluate social resources and activate decision‐making processes (Dworski‐Riggs & Langhout, 2010). The degree of stakeholder interest in community problems and potential actions also influences trust and favorability toward willingness to support new community actions (Wang & Aenis, 2019). Therefore, it is insightful to implement projects that can develop beneficial relationships between different stakeholders, and this is possible through the implementation of interventions within community‐based participatory frameworks. Such interventions actively engage the different stakeholders to promote change and cooperation in dealing with difficult situations, such as those related to a traumatic event in the community (Trickett, 2009). In summary, to promote community resilience, it is necessary to involve community members at all stages, such as partnership development, community assessment, data collection and analysis, dissemination, action and evaluation through participatory research processes that are able to activate reflective learning, a key aspect of building resilience (Ross & Berkes, 2014). In this study, we employed stakeholder analysis and ecological maps to identify the key actors and relationships present in the community following a traumatic event. Our work is situated in the initial stages of the community‐based participatory research (CBPR) framework, where the focus is on building relationships between researchers and stakeholders, as well as identifying the community's resources. CBPR promotes collaborative and empowering relationships between community members, academic researchers and organizations, addressing relevant issues through an ecological health model (Israel et al., 1998; Sánchez et al., 2021). This framework addresses the complexities of stakeholder relations, promoting collective learning and community decision‐making, ultimately leading to community empowerment and deeper connections between researchers and participants (Case et al., 2014; Israel et al., 2012; Wallerstein & Duran, 2006). Unlike traditional “top‐down” disaster responses, CBPR advocates a “bottom‐up” approach that leverages local partnerships, which are essential for community resilience (Chandra et al., 2013; Ellis & Abdi, 2017). This method strategically combines the strengths of stakeholders to effectively address community resilience, as demonstrated by numerous studies (Gagnon et al., 2016; Madsen & O'Mullan, 2016; Migliorini et al., 2023).

## THE CASE OF MORANDI BRIDGE COLLAPSE

**Figure 1:**
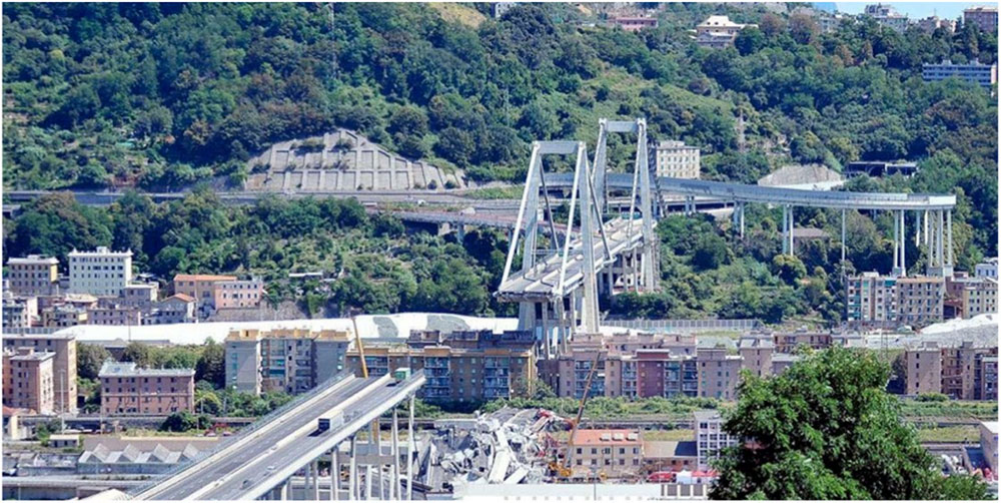
Morandi Bridge disaster in Genoa, Italy.

On August 14, 2018, a tragic and unexpected event occurred in Italy (Figure 1). In the city of Genoa, a highway bridge called Ponte Morandi collapsed without warning due to poor maintenance, claiming 43 lives. This collapse had economic, social and emotional repercussions. More than 250 families had to leave their homes because they were inaccessible or at risk for months. In addition, the city had major problems with the road system and traffic in the following years, leaving some neighborhoods isolated for long periods of time. This tragedy hit the population of the two neighborhoods most affected by the bridge collapse the hardest; moreover, the event took the form of a community trauma at the extended community level.

In the initial phase of an emergency, psychologists and psychotherapists volunteered to support the directly affected population. In the following years, the Local Health Service conducted psychological support work to help the population deal with their traumatic experiences. The description of these interventions shows the feelings of anger, distrust, frustration, fear and trauma experienced by those directly affected by the collapse, the relatives of the victims and the residents of the neighborhoods (Romeo et al., 2020). In a study with citizens, Rania et al. (2019) found that participants expressed feelings of emptiness, helplessness and fear due to the traumatic shock. The collapse of the bridge, both in its literal and symbolic meaning, seemed to have affected the entire community. It brought despair and distrust to the institutions responsible for protection and safety and led to the loss of spaces that many considered routine, compounding the psychological impact of the trauma. These psychological responses are consistent with other studies (Mason et al., 2010) looking at trauma associated with environmental disasters such as flooding. These traumatic experiences lead to a sense of loss of identity and hope for the community. In addition, people may develop stress‐related symptoms, anxiety and adjustment problems that interfere with the proper functioning of the individual and the community. This can have negative psychological consequences, such as the inability to identify one's sense of self with the physical location of home (Makwana, 2019; Romoli et al., 2022).

In 2020, the Social Policy Department along with the Community Service Team Group, the Therapeutic Intervention Group, the Regional Council of Psychologists, and academic researchers sponsored the *Beyond the Bridge* Project to create a dedicated space in the trauma‐affected geographic area for citizens and associations to facilitate and promote active citizenship experiences and community resilience, and to provide psychological and social support.

The project included the creation of community spaces, including a “neighborhood house” where citizens could participate and initiate group activities. Extracurricular and social activities are organized in these spaces, as well as family workshops, computer courses for the elderly, neighborhood services counseling centers, psychological support for individuals and groups, and a co‐working space specifically for young entrepreneurs and start‐ups. Various associations and informal groups have been engaged and coordinated by the Project Lead Group (PLG) for space management, dissemination of initiatives and network building between different local institutions. Action plans include community engagement, partnerships with stakeholders and fundraising initiatives. The project also includes the revitalization of certain urban areas. One of these is called “Clearing of Memory” and is dedicated to initiatives and commemorative events related to the tragedy as well as community‐building activities for residents. These community actions aim to foster connections and social capital, strengthen emotional bonds between community members, and support the sharing of resources and information within the community (Patel et al., 2017).

Existing research has shown the importance of assessing resilience around the Morandi Bridge using an index that considers the role of the university, municipality, residents and industry (Candia et al., 2020). Community resilience, in particular, was found to be the most appropriate construct for this context, as the trauma affected the entire community and created a sense of shared experience that can be managed through community involvement on multiple levels (Rania et al., 2019). Post‐trauma projects need to occur at multiple levels (individual, family, community) and evolve over time as needs change (Norris & Stevens, 2007). A review study (Tariq et al., 2021) found that many methods for assessing community resilience use multiple approaches and emphasize the importance of participatory action. Involving local stakeholders takes the form of a participatory approach and can lead to a better understanding and promotion of community resilience in contexts that have experienced trauma.

Furthermore, a participatory approach seems appropriate to obtain more information. In this context, the Communities Advancing Resilience Toolkit (CART) contains theory and evidence‐based tools that can be used in trauma‐affected communities to improve community resilience by bringing stakeholders together to address community issues (Pfefferbaum et al., 2013). The various CART tools, such as stakeholder analysis or ecological maps, that promote participation, connection, critical reflection, and communication among community members can be used to strengthen community resilience in the aftermath of traumatic situations such as Covid‐19 or in communities characterized by collective trauma (Shigemoto, 2021). Through a shared understanding of the complex relationships between the various community actors, the ecological map contained in CART can facilitate effective decision‐making and planning, leading to improved community resilience as a whole (Pfefferbaum et al., 2013).

Based on the framework outlined above, the aim of the present study is to provide researchers and practitioners with a model for applying CART, within the (CBPR) framework, in a trauma‐challenged context that aims to promote community resilience through the involvement and activation of local stakeholders at all stages of the project.

In this article, the authors look at a case study of a partnership established with a post‐traumatic community in Genoa as part of the *Beyond the Bridge* Project to build community resilience. The study draws on the findings of Gagnon et al. (2016) and utilizes the CART intervention, in line with the CBPR framework.

## METHOD

In this study, CART methodology was implemented through cyclical process steps such as creating partnerships, defining the problem, implementing actions and reflecting on the actions (Pfefferbaum et al., 2013). Community stakeholders, organizations, and individuals from the community built partnerships with researchers by co‐designing the study and its objectives to improve the health and well‐being of the community.

The process following the CART comprised the following steps: a preliminary meeting was held in October 2020 to present the *Beyond the Bridge* Project, during which the PLG was formed. The PLG included representatives from the following groups: the Community Service Team, Social Policy Department Group, Therapeutic Intervention Group, Regional Council of Psychologists, and academic researchers. These groups, as promoters, were naturally invested in the implementation of the project and driven by a common interest in the program, chose to participate independently in the different steps of the project. The PLG oversaw collaborative planning and problem‐solving activities and participated in regular monitoring conference calls. The PLG tracked the entire course of the project through monthly coordination, follow‐up, and review meetings.

Then, a stakeholder analysis was conducted. It is a technique used to identify stakeholders who may influence interests with respect to initiative, power, awareness, and favorability. It can also be used to develop strategies to enhance support and limit opposition towards postdisaster interventions implemented in the territory by anticipating reactions. Significant organizations present in the territory affected by the trauma, identified by the PLG as stakeholders in the *Beyond the Bridge* Project, were considered. The stakeholder analysis tool from CART was used. The tool is represented by a chart comprising the constructs of interest, awareness, power and favorability. To perform the analysis, 17 members of the PLG completed an online questionnaire wherein they indicated for each stakeholder present in the territory affected by the trauma and not included in the PLG, the degree (assigning a score on a Likert scale from 0 “very disinterested” or “unaware of initiative” or “not powerful” or “strongly opposes” to 5 “very interested” or “very aware of initiative” or “very powerful” or “strongly favors”) of interest (understood as the specific benefits, costs, and changes that will result from your project), awareness (understood as the extent to which stakeholders know about your project), and power (understood as the influence that stakeholders have in the adoption and implementation of the project), favorability (understood as stakeholder attitude toward your project) with respect to the *Beyond the Bridge* Project, as perceived by the members of the PLG.

Subsequently, community ecological maps (ecomaps) were used. The ecological map of a community is an important visual tool for understanding the relationships between its members and groups. It is useful for identifying and improving difficult relationships with stakeholders, identifying and developing potentially beneficial partnerships, and obtaining a complete picture of the interaction dynamics present in areas where the *Beyond the Bridge* Project has been implemented. An ecomap for the community was developed following instructions provided by Pfefferbaum et al. (2013). The instructions for creating the ecomap were: “Write the name of your organization in the large central circle. Draw circles representing the identified stakeholders with whom you currently interact. The size of the other circles should reflect the frequency or level of interaction with your organization. The strength of your relationship with each group is described by connecting lines from your organization to the others”. The academic researchers, as PLG coordinators, asked representatives of the Community Service Team, Social Policy Department Group, Therapeutic Intervention Group, Regional Council of Psychologists groups to complete their ecological map in separated subgroups. Key organizations present in the trauma‐affected area and identified as stakeholders by the PLG were considered, and the frequency with which each subgroup of the PLG interacted with them was assessed. For this purpose, a circle was drawn to represent the PLG subgroup and other circles were drawn to represent the stakeholders. It is not their location that is important, but their size, which should reflect the frequency or level of interaction with the PLG. PLG members were then asked to indicate the strength and nature of these relationships. A solid line indicated a strong and positive relationship, a dotted line indicated a weak and positive relationship, and a zigzag line indicated a stressful or problematic relationship. In each subgroup, the representatives discussed and shared their perspectives on the issues at hand. Afterward, they selected one member to document the group's collective insights on the ecomaps. These insights were then shared with the rest of the PLG. Table 1 details the different stages involved in the application of the Cart in line with the CBPR framework.

**Table 1:** Overview of the process of application of CART within CBPR framework.

Aims	Description
*First Meeting—Date: 8 October 2020—Location: Morandi Bridge Neighborhood*	
PLG constitution	The client (Social Policy Department) presents the postdisaster community project (*Beyond the Bridge Projec*t) to all interested partners. University community researchers present the objectives and functions of the PLG.
*Second Meeting—Date: 28 October 2020—Location: Morandi Bridge Neighborhood*	
Develop constructive working relationships in PLG and maintain them over time	Participants share project keywords and overarching objectives and describe their potential roles in the project. During the discussion, Lead Group members listen to and consider others' viewpoints. The project logo has been identified.
*Third Meeting—Date: 9 December 2020—Location: Morandi Bridge Neighborhood*	
Seek opportunities to improve project communication and dissemination	Project dissemination initiatives (letters to institutions, flyers) were discussed, and an action plan was developed. The PLG planned the tasks to implement the project.
*Fourth Meeting—Date: 11 February 2021—Location: Morandi Bridge Neighborhood*	
Identify and prioritize the key stakeholders involved to facilitate or hinder the project development through stakeholder analysis	Stakeholders were identified by the PLG in a process that was divided into two stages: initially, all group members made a list of stakeholders. Then, through a group discussion, the main ones were identified. Subsequently, a stakeholder analysis was conducted. Selected stakeholders' attitudes towards the post‐trauma intervention are provided, using the variables interest, awareness, power and favourability according to the CART tool.
*Fifth Meeting—Date: 23 March 2021—Location: Morandi Bridge Neighborhood*	
Identify primary, secondary and key stakeholders	PLG members discuss to understand if identified stakeholders could be influenced by whom and by what within the context.
*Sixth Meeting—Date: 29 April 2021—Location: Morandi Bridge Neighborhood*	
Build and discuss ecomaps to improve relationships and partnerships	Using the visual tool of Ecomaps, PLG describes the nature and strength of community relationships. Participants work in subgroups and discuss together the potential benefits and costs of increasing the frequency of interaction with some organizations and ways to reduce stress in problematic relationships.
*Seventh Meeting—Date: 20 May 2021—Location: Morandi Bridge Neighborhood*	
Collect information that can help to match community resources and capabilities with the environment	Participants identified key stakeholders and discussed how to connect with them to engage them in a qualitative interview. Academic researchers carried out interviews.
*Eighth Meeting—Date: 17 June 2021—Location: Morandi Bridge Neighborhood*	
Collect information that can help to match community resources and capabilities with the environment	Participants identified key stakeholders and discussed how to connect with them to engage them in a qualitative interview. Academic researchers carried out interviews.
*Ninth Meeting—Date: 18 November 2021—Location: Morandi Bridge Neighborhood*	
Develop appropriate strategies and solutions for dealing with weaknesses and threats	PLG set goals for the issues they choose to address and participates in planning strategy development for enhancing community resilience in support of their established objectives
*Tenth Meeting—Date: 13 December 2021—Location: Morandi Bridge Neighborhood*	
Develop appropriate strategies and solutions for dealing with weaknesses and threats	PLG set goals for the issues they choose to address and participates in planning strategy development for enhancing community resilience in support of their established objectives

*Note*: In any step, the PLG discuss the assessment, feedback, planning, and action about the postdisaster project.

Abbreviations: CART, Communities Advancing Resilience Toolkit; PLG, Project Lead Group.

## RESULTS

The PLG comprised 17 people from the Community Service Team (*n* = 4), Social Policy Department Group (*n* = 4), Therapeutic Intervention Group (*n* = 5), Regional Council of Psychologists (*n* = 2), and academic researchers (*n* = 2).

The Community Service Team is made up of members of cooperatives formed after the bridge collapse; the Social Policy Department Group is a public body involved in planning and coordinating activities to support families, young people and people with disabilities and to combat social exclusion; the Therapeutic Intervention Group is made up of psychologists who carry out psychotherapeutic interventions using a trauma‐informed approach; the Regional Council of Psychologists is a public body that brings together and guides psychologists in their profession; the academic researchers is made up of academic researchers with expertize in community psychology who serve as the PLG coordinators.

Between the meetings outlined in Table 1, the PLG representatives participated in online briefings and subgroup meetings led by academic researchers to monitor the progress of the *Beyond the Bridge Project*. These online meetings were specifically reserved for the PLG and were scheduled internally among the members. Data and information circulate from one phase to the next as participants build on and complement previous phases. At each step of the process, members could understand and contribute.

### Stakeholder analysis

The 17 members of the PLG identified the following stakeholders: City Hall, Social Services, Schools, Local Health Agency, Family Service Center, Those of the Bridge Association, Free Citizens Committee, Orange Zone Committee, Over the Bridge Association, National Association After Railway Work, LINK Association, Merchant's Associations, and the Parishes. The identification of interest groups is useful for carrying out analyses and ecological mapping. Table 2 presents a list of the different stakeholders with a description of each.

**Table 2:** Description of each stakeholder in the Beyond the Bridge Project.

Stakeholder	Description
City hall (area 2 and area 5)	Administrative decentralization in municipalities
Parishes	Ecclesiastical territorial districts
Social service (area 35 and area 41)	It is an inter‐municipal aggregation whose task is to plan and schedule the social services of the municipalities.
Schools	Neighborhood schools
Those of the Bridge Association	Association established following the bridge collapse
Orange zone committee	Association established following the bridge collapse
Free citizen committee	Entity pursuing a public benefit purpose
National Association after railway work	Free time enterprise
Merchants' association	Association representing traders
Association over the bridge	Committee set up following the bridge collapse
Local health agency	Regional public institution whose purpose is to provide health services
Family service center	Cooperative providing educational activities for minors
LINK association	Association established following the bridge collapse

**Figure 2:**
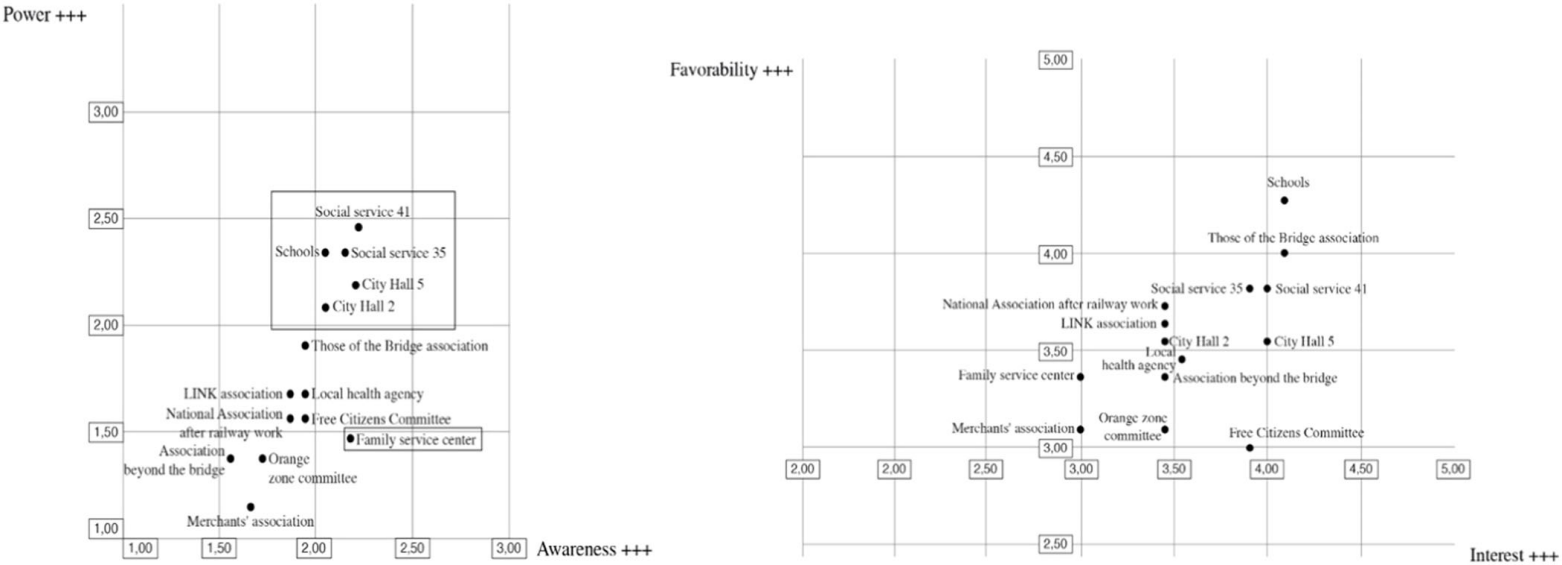
Stakeholder analysis on power and awareness, favorability and interest.

After identifying the community stakeholders, the analysis was conducted using the constructs of power, awareness, favorability, and interest in relation to the *Beyond the Bridge* Project. Figure 2 shows the results of the stakeholder analysis in terms of power and awareness and the results of the stakeholder analysis results for favorability and interest.

## FOLLOWING THIS ASSESSMENT, THE STAKEHOLDERS WERE PRIORITIZED

A power/interest/favorability and awareness grid was used to analyze stakeholder attitudes towards the *Beyond the Bridge* Project. This is an approach designed to create a special space for citizens and associations in the geographical area affected by the collapse of the Morandi Bridge to promote community resilience and provide psychological and social support.

## THE RESULTS OF THE ANALYSIS DIVIDE THE PARTICIPANTS INTO SEVERAL GROUPS

The group of potential promoters includes actors with great power, great interest, favorability, and great awareness of the *Beyond the Bridge* Project*:* City Hall, Social Services, and Schools.

Potential opponents of the project are stakeholders with a lot of power and interest, but little favorability: the members of the Bridge Association and the Committee of Free Citizens.

The stakeholders who are apathetic to the *Beyond the Bridge* Project are the Civic Associations and Family Service Centers.

These findings are interesting because they can provide valuable information on how to effectively address them and build stronger relationships and who can become more involved to promote greater community competence and empowerment, which are key aspects of community resilience. These findings help the PLG to categorize stakeholders and develop effective communication strategies for each category according to their authority (power) and interest in the project outcomes (interest).

Given the importance of these key figures to the geographical area exposed to the Morandi Bridge Collapse, the PLG sought to understand important influencers. From the discussion, it emerged that the school is a powerful stakeholder demonstrating substantial influence. It is not only influenced by “external” figures, such as the cooperatives and the families, but also directly by other stakeholders. A relationship of mutual influence was identified between the two municipalities (Area 2 and Area 5), while there appears to be greater independence between the two social services (Area 35 and Area 41).

## BASED ON THE ANALYSIS OF THE LEVELS OF INFLUENCE, THE FOLLOWING CAN BE STATED

The primary stakeholders are the beneficiaries or recipients of the *Beyond the Bridge* Project. These were identified as schools, cooperatives, and families. Secondary stakeholders (i.e., those whose work or lives could be affected by the project's process or results) were health services, social services, social policies, and city halls in our case. Finally, key stakeholders are those who can most influence others and are interested in the outcome of the *Beyond the Bridge* Project, including government officials, policymakers, and associations.

### Ecomaps

Ecomaps, as described in the CART, were constructed from different subgroups of the PLG (Community Service Team, Social Policy Department Group, Therapeutic Intervention Group, and the Regional Council of Psychologists) to understand the relationships between the groups and other stakeholders.

Ecomaps were created for four groups: Social Policy Department Group, Community Service Team, Psychotherapeutic Team, and Regional Council of Psychologists. The ecomap from Social Policy Department Group represented many relationships but with weak ties. In fact, the ecomap shows discontinuous relationships with different stakeholders such as with the local health agency and also reveals a conflicting relationship with City Hall 5. In addition, from the size of the circles, a higher frequency of interaction can be observed with institutional bodies since the City Hall circles are larger. The ecomap of the Community Service Team shows constant interactions with National Association of After Railway Work and the LINK Association supporting neighborhood initiatives. Discontinuous relationships with those of the Bridge Association, Social Service area 41 and 35, Schools and Town Hall area 2 and conflictual relations with City Hall area 5, Committee Free Citizens and Municipality of Genoa. No relations are represented with other stakeholders. Also in this map, the largest circles, representing the highest frequency of interaction, mainly concern institutional bodies such as the City Hall and the Municipality. The ecomap of the Psychotherapeutic Team represents few relationships in the community. In particular, constant relations with School of Certosa, those of the Bridge Association and Social Service 41 and conflictual relations with School of Rivarolo with which they have less interaction as indicated by the size of the circles. Finally, the ecomap of the Regional Council of Psychologists reveals almost no relationship within the community, except for discontinuous relations with schools and positive relations with Local Health Agencies although they are not very frequent.

**Figure 3:**
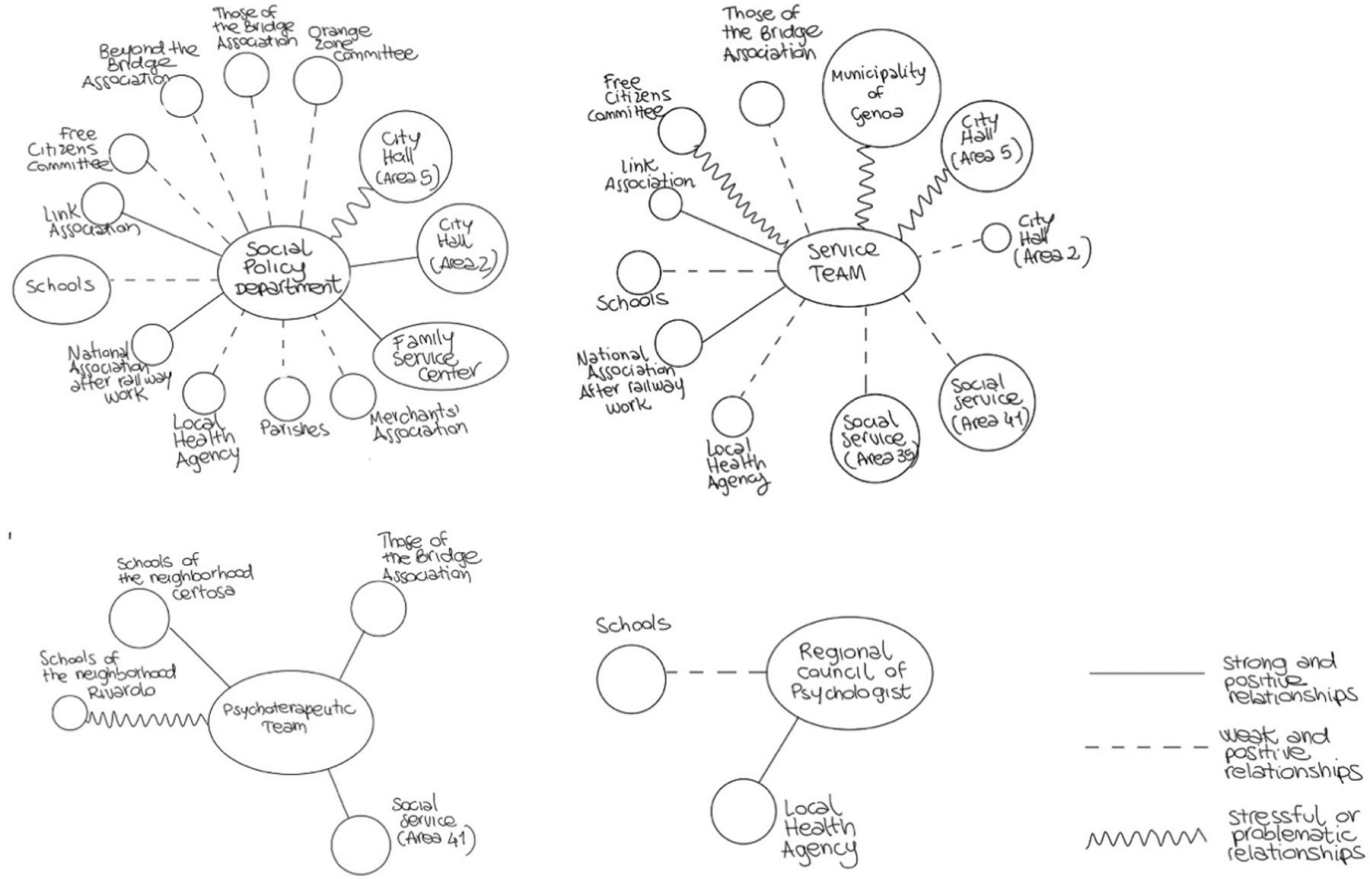
Ecomaps made by Social Policy Department, Community Service Team, Psychotherapeutic Team, Regional Council of Psychologist.

Figure 3 presents the ecomaps of the PLG subgroups. Each image was created by each team (the Department of Social Policy, the Service Team, the Psychotherapeutic Team and the Regional Council of Psychologists) and shows how each group perceives the relationships surrounding it. Each ecomap was created through a prior discussion between the different members of the group. Once a common agreement was reached, the ecomap was created by a representative of the group, chosen by the group itself under the supervision of the other members.

The examination of the ecomaps made it possible to identify several organizations that were not yet involved; in particular, PLG points to municipal educational services for the 0–6 age group (crèches and kindergartens), as well as associations focused on older adults and citizens, both as community groups and as unorganized individuals. The Regional Council of Psychologists emphasized the importance of reaching out to cultural, recreational and sports associations within a community. Accordingly, it is also important to increase the frequency of interactions with various stakeholders to raise the visibility of the psychology profession and promote community health. Additionally, the participants highlighted the limits to the expansion of interactions, which mainly concerned the working and organizational time of the Social Policies Department, and the complexity caused by the increased amount of work involved. All groups emphasized the need to improve public relations, which aimed to make the *Beyond the Bridge* Project known in the area and create goodwill, and internal communication, with the intention of developing involvement and cooperation among participants.

## DISCUSSION

This study provides researchers and practitioners with a model for applying CART within a CBPR framework (Wallerstein & Duran, 2006) in a trauma‐affected context, aiming to promote community resilience through the involvement and activation of local stakeholders throughout all phases of the project. The study is based on a community response to trauma—the *Beyond the Bridge* Project—to promote community resilience in the context of the trauma related to the collapse of the Morandi Bridge in August 2018. Following this trauma, the affected community experienced feelings of hopelessness and fear towards institutions. To overcome this trauma and its consequences, promoting community resilience is central as it supports the community to promote resources and reduce the impact of the trauma (Tariq et al., 2021). Through the implementation of this project, it was possible to foster communication, reflection, community competence in collective action, and the ability to solve problems and make decisions, leading to collective effectiveness and empowerment. Placing our findings in the broader context of the community resilience process (Matarrita‐Cascante et al., 2022; Norris et al., 2008), it is important to recognize that the *Beyond the Bridge* Project and our approach to CBPR contribute valuable insights to the ongoing discourse on community resilience. Our study highlights the dynamic nature of community resilience following trauma and emphasizes the importance of fostering emotional connections, communication, and community competence.

We implemented the CART methodology, within the CBPR framework, according to Gagnon et al. (2016) to assess and promote a post‐traumatic intervention through the creation of a PLG comprising various stakeholders such as social policy members, academic researchers, and therapeutic intervention groups. In this study, the term “community” denotes the “Beyond the Bridge Project” and its associated project actors, including stakeholders. This collaborative effort, initiated by PLG, involved a series of extensive and long‐term processes. This included stakeholder identification and stakeholder analysis that was aimed at assessing the impact of stakeholders on the project, and interrelationship analysis between stakeholders utilizing ecological maps to identify key stakeholders.

PLG was involved in the evaluation and implementation phase of the project. This finding is in line with Pfefferbaum et al. (2013), who argue that a participatory approach to assessing and promoting community resilience is appropriate as it allows for the involvement of local stakeholders, who are ideally engaged throughout the process. The involvement of stakeholders at each stage of this application enabled the promotion of community competence in terms of a sense of working towards a common goal, critical reflection and the promotion of community ownership, which are key aspects of community resilience (Norris et al., 2008). In their review study, Tariq et al. (2021) also emphasize the importance of using participatory measures in promoting community resilience.

The utilization of the CART enhances community engagement and resilience. Regarding the involvement of stakeholders after trauma, the literature points out that they are mostly involved in the needs assessment phase and less involved in the decision‐making process (Cavanaugh & Wismar, 2022). Through the analysis of stakeholders and the creation of ecomaps, a collaborative process was activated in which all members were involved in an active and productive confrontation in which thoughts, ideas, procreations and suggestions were shared. These tools allowed the members involved in the project to feel competent and to establish an effective communication style, key aspects of community resilience.

Stakeholder engagement promotes a sense of community ownership and engaging stakeholders in creating effective partnerships between communities and organizations promotes community resilience (Jewett et al., 2021). The results of the stakeholder analysis in this study show that the PLG was able to effectively identify and categorize key stakeholders based on their authority (power), interest (concern), benevolence, and awareness of the *Beyond the Bridge* Project. The categorization of stakeholders into potential supporters, potential opponents and those who are apathetic to a project can be used to develop effective communication strategies for each group to enable targeted engagement with key stakeholders. It is also possible to implement strategies that engage stakeholders who are apathetic to the project, for example, through interventions to promote interest in the project that results from actively engaging these stakeholders and making them active protagonists of the community. This approach is in line with the literature (Cunningham‐Erves et al., 2020) on stakeholder analysis, which emphasizes the importance of identifying key stakeholders for the effective planning and implementation of community‐based interventions.

The project also used ecomaps to identify the relationships between stakeholders and understand how they influence each other. This approach is consistent with the community ecology literature (Wu & Chen, 2023), which posits that understanding the relationships between the different actors in a community is key to understanding and addressing complex community problems.

The stakeholder analysis and ecological maps, integral components of CART, not only provide a nuanced understanding of stakeholder dynamics but also illuminate the evolving relationships within the community post‐trauma. The ecomaps used in this study suggest that different subgroups within the PLG have varying levels of relationships and interactions within the community.

However, we must consider that, despite the existence of a network of sometimes weak and conflictual relationships among the present partners in the territory, there is a lack of connection with the new subjects emerging due to the trauma. After a trauma, new social actors enter the community and must relate to the social actors already present in the community. Therefore, it is essential to foster relationships with other social actors and to utilize the skills of the new actors in the community. Stakeholder identification, analysis and ecomapping show that spontaneous connections emerge in response to a traumatic event (e.g., numerous associations are formed after a bridge collapse); however, external responses to the tragedy and its drivers must also be incorporated into the network. To foster community resilience after a traumatic event, it is important to strengthen the connections between different institutions in the area. Additionally, engaging community stakeholders through a participatory approach is essential for enabling this (Ellis & Abdi, 2017). Ecomaps allow us to identify organizations that lack potential and limit the expansion of interactions. This information can be beneficial for PLG to develop effective communication and strategies to involve more community members and organizations in the project.

Consequently, our study highlights the significance of engaging both established and new social actors in the aftermath of a traumatic event. The identification of spontaneous connections and the integration of external responses into the community network underscores the importance of adaptability and inclusivity in fostering community resilience.

This study is consistent with previous research that emphasizes the importance of community engagement and stakeholder analysis in promoting community resilience. For example, Wells et al. (2013) found that effective community engagement strategies for disaster risk reduction involve building trust and relationships with stakeholders and involving community members in planning and implementing actions. In another study (Silberberg & Martinez‐Bianchi, 2019), stakeholder engagement was found to be a critical aspect of successful CBPR to promote resilience, as it enables shared decision making and buy‐in from key stakeholders.

To summarize, the results of the ecomaps and stakeholder analysis indicate that the PLG had varying degrees of relationships and interactions within the community, with some subgroups having stronger ties than others. The ecomaps and stakeholder analysis can be useful in identifying uninvolved organizations and developing effective communication strategies to engage more community members and organizations in the project to promote community resilience. In addition, the use of these tools provides the opportunity for effective discussion, dialogue, and communication between different community stakeholders, which helps to overcome the skepticism that the community often exhibits when working with, for example, academics, as Mance et al. (2020) point out.

Finally, our research expands the understanding of community resilience by demonstrating how CART, specifically employing the CBPR framework, can effectively promote community resilience in the context of collective trauma. The successful application of ecomaps and stakeholder analysis in the *Beyond the Bridge Project* suggests transferability to other post‐traumatic settings. For instance, these tools can be adapted for use in communities recovering from natural disasters, industrial accidents, or social upheavals, providing a comprehensive understanding of post‐trauma dynamics. Moreover, the adaptability of these methodologies allows for effective use also in communities with different cultural backgrounds, acknowledging the universal principles of community resilience while accommodating specific contextual nuances.

There are several limitations in this paper, however. First, we presented data related to data collection conducted in a specific period, which may limit the ability to track changes over time or identify long‐term trends in community resilience. A long‐term follow‐up study could further enrich the understanding of post‐traumatic dynamics in the community. In addition, the study focused on stakeholder engagement, community competence, and effective communication. The study did not address other factors that may impact community resilience, such as access to resources.

## CONCLUSION

The tragic events surrounding the collapse of the Morandi Bridge on August 14, 2018, claimed 43 lives. It shocked the city and the nation, prompting the public and private sectors to promote not only support in the emergency phase, but also interventions to strengthen community resilience and bring about medium and long‐term change. The findings have implications for using a CART in community resilience, stakeholder engagement, and community action leadership. The framework provides a bottom‐up approach to promoting community resilience and fostering the necessary collaboration between researchers and community members.

This project highlights the benefits of including the perspectives of different stakeholders in the research cycle, including academic, community and socio‐political partners. In this context, particular attention should be paid to the unique contributions of each research partner, and the relationships between partners must be considered to promote the effectiveness of the *Beyond the Bridge* Project over time.

By analyzing community response to trauma, this study has shown that CART can be used to promote community resilience by engaging diverse social actors to support community competence and effective communication that requires participation in individual and collective meaning‐making processes related to traumatic events. When social research aims to address trauma in the community, stakeholder analysis offers practical models for engaging the community and increasing the project's chances of success. The findings presented here suggest that it is important to understand the role of stakeholders in the processes of socio‐ecological and institutional change following trauma.
